# Gas exchange and leaf anatomy of a C_3_–CAM hybrid, *Yucca gloriosa* (Asparagaceae)

**DOI:** 10.1093/jxb/erv536

**Published:** 2015-12-29

**Authors:** Karolina Heyduk, Nia Burrell, Falak Lalani, Jim Leebens-Mack

**Affiliations:** ^1^2502 Miller Plant Sciences, Department of Plant Biology, University of Georgia, Athens, GA 30602, USA

**Keywords:** Crassulacean acid metabolism, gas exchange, hybrid, leaf anatomy, photosynthesis, physiology.

## Abstract

While the majority of plants use the typical C_3_ carbon metabolic pathway, ~6% of angiosperms have adapted to carbon limitation as a result of water stress by employing a modified form of photosynthesis known as Crassulacean acid metabolism (CAM). CAM plants concentrate carbon in the cells by temporally separating atmospheric carbon acquisition from fixation into carbohydrates. CAM has been studied for decades, but the evolutionary progression from C_3_ to CAM remains obscure. In order to better understand the morphological and physiological characteristics associated with CAM photosynthesis, phenotypic variation was assessed in *Yucca aloifolia*, a CAM species, *Yucca filamentosa*, a C_3_ species, and *Yucca gloriosa,* a hybrid species derived from these two yuccas exhibiting intermediate C_3_–CAM characteristics. Gas exchange, titratable leaf acidity, and leaf anatomical traits of all three species were assayed in a common garden under well-watered and drought-stressed conditions. *Yucca gloriosa* showed intermediate phenotypes for nearly all traits measured, including the ability to acquire carbon at night. Using the variation found among individuals of all three species, correlations between traits were assessed to better understand how leaf anatomy and CAM physiology are related. *Yucca gloriosa* may be constrained by a number of traits which prevent it from using CAM to as high a degree as *Y. aloifolia*. The intermediate nature of *Y. gloriosa* makes it a promising system in which to study the evolution of CAM.

## Highlight

Physiological and anatomical study of a C_3_–CAM hybrid species, which has the ability to use CAM under drought and has significant anatomical differences from both C_3_ and CAM parental species.

## Introduction

The daily fixation of atmospheric carbon dioxide is a defining trait of green plants, and is arguably the basis for the major part of terrestrial biodiversity. Plants experience a number of stresses that make the photosynthetic machinery less than optimal, including drought, shade, and high temperatures. As they cannot relocate to avoid the stress, plants have instead modified their photosynthetic pathways in ways to circumvent limitations caused by abiotic stress. One such modification, Crassulacean acid metabolism (CAM), has evolved a number of times independently across angiosperms in response to carbon limitation due to water stress. Most plants that use the C_3_ carbon metabolism pathway fix CO_2_ during the day via the carboxylation of ribulose-1,5-bisphosphate by the enzyme Rubisco. However, Rubsico has both carboxylase and oxygenase activity, the latter of which is favoured in high temperatures or under low CO_2_ levels, resulting in costly photorespiration. For C_3_ plants during times of water stress, stomata close to prevent water loss, which leads to a depletion of internal CO_2_ and initiation of photorespiration. To simultaneously increase carbon uptake while minimizing water loss, CAM plants instead open their stomata at night, when transpirational rates are lowest. Atmospheric CO_2_ is converted to a four-carbon intermediate in the form of malic acid and stored in the vacuole until day time, when stomata close and the malic acid is decarboxylated (Osmond, 1978; Winter and Smith, 1996). The resulting internal concentrations of CO_2_, which is fixed by Rubisco behind closed stomata, are as high as 2% (Cockburn *et al.*, 1979), compared with 0.38–0.4% outside the leaf. CAM species have estimated water use efficiency (moles of CO_2_ fixed per mole H_2_O transpired) of (6–30)×10^–3^, compared with (0.6–1.3)×10^–3^ for C_3_ plants (Lüttge, 2004).

CAM is found in about 6% of flowering plants and is distributed across 35 plant families (Cushman, 2001; Silvera *et al.*, 2010). CAM species share a suite of physiological characteristics, including the ability to fix carbon at night, the accumulation of malic acid in the vacuoles over the dark period, increased leaf succulence due to enlarged vacuoles for the storage of malic acid, and daily carbohydrate turnover for the regeneration of phospho*enol*pyruvate (PEP), the molecule carboxylated for the initial fixation of carbon by the enzyme PEP carboxylase (PEPC). Leaf anatomy has also been implicated in the evolution of CAM, with previous research indicating tight cell packing (or low intercellular airspace; IAS) and large mesophyll cells as requirements for the optimal CAM function (Nelson and Sage, 2008; Zambrano *et al.*, 2014). Changes in these two traits may be linked responses to selection for increased vacuolar storage of malic acid in CAM species, but this hypothesis needs to be tested. Vein density has been shown to be a critical trait for the evolution of C_4_ photosynthesis and is central to Kranz anatomy (Hattersley, 1984; Ueno *et al.*, 2006; McKown and Dengler, 2007; Christin *et al.*, 2013), but venation has been largely overlooked in anatomical studies of CAM plants. Interveinal distance has been found to correlate positively with succulence (Ogburn and Edwards, 2013). In addition, stomatal densities in *Clusia* were found to be lower in plants with higher night-time carbon uptake (Zambrano *et al.*, 2014). In general, it is thought that there are tradeoffs between CAM-promoting traits and efficient C_3_ photosynthesis. The optimal anatomy for CAM plants includes large cells and a decrease in the amount of IAS; these same traits would limit efficient conductance of gas throughout the leaf (Nelson *et al.*, 2005; Nelson and Sage, 2008; Zambrano *et al.*, 2014).

Despite the extremes in phenotypes, CAM is often described as a continuum or spectrum, with full CAM at one end, C_3_ at the other, and various intermediate forms between (Winter *et al.*, 2015). Although CAM plants are defined by night-time carbon uptake, this dogma ignores the high degree of plasticity found in CAM plants (Dodd *et al.*, 2002). Some CAM lineages have ‘weak’ CAM plants, predominantly C_3_ plants with very low levels of CAM expression (as measured by leaf titratable acidity) (Silvera *et al.*, 2005). In addition, some species, including those in *Clusia* and *Mesembryanthemum*, are known to be facultatively CAM, whereby they use the C_3_ pathway under non-stressed conditions but can upregulate the CAM cycle in response to a variety of abiotic stresses. Detailed comparative studies between C_3_, CAM, and intermediate forms may help bridge the evolutionary gap between the ends of the spectrum (Silvera *et al.*, 2010; Winter and Holtum, 2014; Garcia *et al.*, 2014). Correlations between the ability of intermediate species to use the CAM pathway and their physiology and leaf anatomy could pinpoint traits that are vital—or not—to night-time carbon fixation.

To assess how physiology and anatomy correlate to a species’ ability to use CAM, a natural hybrid system in the genus *Yucca* L. (Asparagaceae) was explored: *Yucca aloifolia* (CAM) and *Yucca filamentosa* (C_3_), which are sympatric in the southeastern USA and have hybridized to form *Yucca gloriosa* (Rentsch and Leebens-Mack, 2012). *Yucca gloriosa* is unlikely to be a recent F1 hybrid (Rentsch and Leebens-Mack, 2012), and this species may be segregating for parental phenotypes as well. The parental species and the hybrid overlap in habitats along the dunes of the southeastern coastline, but grow in different parts of the dune system and are morphologically distinct. *Yucca filamentosa* inhabits the scrub-pine forests behind the dunes, except in the northern parts of its range, where it lives on the dunes under the protection of nurse plants. *Yucca aloifolia* and *Y. gloriosa* are both foredune species, with *Y. gloriosa* typically found on the ocean-side of the foredune. *Yucca gloriosa* was previously shown *in situ* to accumulate significant amounts of malic acid during the night, but showed no concurrent night-time ^14^CO_2_ uptake (Martin *et al.*, 1982). The lack of night-time carbon uptake could be an artifact of sampling methodology used, especially if gas exchange rates are low. Genotypes from each of the three species were collected from across the species’ ranges and assessed for photosynthetic pathway. Plants were assayed for carbon uptake in growth chambers under well-watered conditions, as well as while drought stressed, as CAM has been shown to be up-regulated under drought in various species (Ting, 1985; Lee and Griffiths, 1987; Winter and Ziegler, 1992; Winter *et al.*, 2011). These same *Yucca* individuals were phenotyped for titratable acidity and leaf anatomical characteristics. To better understand what suites of traits are important for CAM function, and how they impact the ability of *Y. gloriosa* to use either photosynthetic pathway, physiological traits were assessed for correlations.

The extent to which the hybrid species *Y. gloriosa* exhibits characteristics of its C_3_ and CAM parents was also evaluated. Phenotypic assessment shows parental species are true to type, displaying gas exchange patterns and leaf anatomical traits that are predicted by their respective photosynthetic pathways. The hybrid species *Y. gloriosa* uses both C_3_ and CAM pathways to assimilate carbon, and converts to fully CAM when drought stressed. Moreover, it is argued that *Y. gloriosa* can serve as a new study system for investigating the genetic architecture and evolution of CAM within the Agavoideae, a group that includes some of the most iconic CAM species.

## Materials and methods

### Plant acquisition and maintenance

All three *Yucca* species are clonal and readily generate ramets from the base of larger maternal plants. In summer 2013, ramets between 10 and 15cm tall were collected from all three species across the southeastern US seaboard, from the Outer Banks of North Carolina to the barrier islands of Georgia (Supplementary Table S1; Supplementary data are available at *JXB* online). Two clonal ramets were collected from each maternal plant and transplanted to the University of Georgia greenhouses in a 50:50 by volume mix of sand:pine bark (with vermiculite and limestone added). Plants were watered and fertilized as needed for at least 9 months prior to experimentation (described below). Diseased plants or those where size dimorphism between clones was very large were excluded.

### Genotyping of individuals

To assess a degree of genetic variation among individuals of all three species, microsatellite markers were used from Flatz *et al.* (2011) or Rentsch and Leebens-Mack (2012), or were developed *de novo* for this study (see Supplementary Table S2). *De novo* development of primers took advantage of unpublished sequence capture data in *Y. aloifolia* (see Heyduk *et al.*, 2015 for an overview of methods). An assembled set of contigs from the sequence capture data was first analysed for a single *Y. aloifolia* individual (Y45) through BatchPrimer3 for microsatellite discovery. These putative loci were then screened in the sequence capture assemblies from an additional five genotypes of *Y. aloifolia*. Polymorphism across all six genotypes was assessed *in silico,* and loci that varied in repeat length across the individuals of *Y. aloifolia* were kept for screening via PCR. A final screening left seven loci from each of the three resources (Flatz *et al.* 2011; Rentsch and Leebens-Mack, 2012; this study) that amplified successfully in all three species.

DNA from individuals used in phenotypic analysis (Supplementary Table S1) was isolated using a modified cetyltrimethylammonium bromide (CTAB) method (Doyle, 1987; Štorchová *et al.*, 2000). DNA was amplified for the microsatellite loci using a three-primer system, where one primer (M13) is fluorescently tagged with either a FAM or HEX fluorophore, and the forward primer has an additional sequence that is complementary to the M13 primer sequence (Schuelke, 2000). Loci were amplified with the following PCR mix: 3.6 µl of PCR buffer (100mM Tris-HCl pH 8.0, 500mM KCl), 0.9 µl of 25mM MgCl_2_, 0.6 µl of 10mM dNTPs, 0.4 µl of reverse primer (10 µM ), 0.4 µl of M13 (10 µM ), 0.375 µl forward primer (2 µM), 6.725 µl of H_2_O, 1 µl of *Taq* polymerase, and 1 µl of template DNA diluted to ~10ng µl^–1^ for a total of 15 µl per PCR reaction. Amplification used a touchdown programme as follows: initial denaturation at 95 ºC for 2min; 10 cycles of 95 ºC for 15s, 64 ºC for 15s with a 1degree drop per cycle, and 72 ºC for 30s; 25 cycles of 95 ºC for 15s, 54 ºC for 15s, and 72 ºC for 30s; a final extension at 72 ºC for 1min. PCR products were diluted 1:15, pooled when appropriate, then 3 µl was mixed with 10 µl of a formamide–ROX dye-labelled size standard (1ml formamide, 100 µl ROX ladder). Fragment analysis was conducted on an Applied Biosystems 3730xl DNA Analyzer. Alleles were called using Geneious 8.1.6 (Kearse *et al.*, 2012), exported, and analysed for hybrid index scores using the introgress package (Gompert and Buerkle, 2010) in R 3.2.2 (R Core Team, 2013).

### Gas exchange

A total of 16 genotypes were phenotyped: three from *Y. aloifolia,* four from *Y. filamentosa*, and nine from *Y. gloriosa*. In order to assess gas exchange patterns on a large number of plants, genotypes from all three species were split between three independent experiments conducted in July and October 2014 and February 2015 (Supplementary Table S1). For a given experimental period, five to six genotypes each with two clones were measured. Genotypes were randomly assigned to one of three time blocks, and individual clones of each genotype were randomly assigned to the water or drought treatment. Plants were moved into the growth chamber 1 week prior to the onset of any experimental treatment and all plants regardless of assigned treatment were watered daily. Growth chamber conditions were set to day/night temperatures of 32/17 °C, with a relative humidity of 30% and day length of 12h. Photosynthetically active radiation (PAR) at plant level was between 400 and 500 µmol m^–2^ s^–1^.

Leaf gas exchange measurements were conducted with a LiCOR-6400XT portable photosynthesis system (LiCOR, Lincoln, NE, USA). The first set of measurements was taken while soil for all plants was still at field capacity (day 1). Gas exchange measurements were taken every 4h for 24h, beginning 1h after the onset of light in the growth chamber. After the initial 24-h interval of gas exchange measurements under well-watered conditions was complete, water was withheld from the drought-treatment plants to initiate a dry-down, while well-watered treatment plants continued to be watered daily. Soil moisture probe measurements taken every other day were used to ensure a relatively even dry-down (Supplementary Table S3). Well-watered and drought-treated plants were then measured for gas exchange rates every other day for three 24-h intervals (days 3, 5, and 7). For each measurement, plants were measured in the same order and the same leaf was used for the gas exchange measurements, unless the leaf showed signs of damage from the LiCor chamber. After the fourth 24-h interval (day 7) of gas exchange measurements were complete, all plants were watered, and a final day of gas exchange measurements was conducted 1 day later (day 9).

### Leaf titratable acidity

Discs of leaf tissue were taken from plants 2h before lights turned off in the evening (p.m.) and two hours before lights turned back on in the morning (a.m.). Samples were taken on the initial day while all plants were well watered (day 1), on two of the three drought-treatment days (days 5 and 7), and on the final re-watered day (day 9). Because of limited tissue, two to three disc samples per plant were taken. Tissue was immediately frozen in N_2_ and stored at –80 °C until leaf titrations commenced. Titrations were done on each sample independently by first measuring frozen weight, then boiling for 20min in 20ml of H_2_O. Boiled samples were allowed to cool to room temperature, then titrated with 100mM NaOH to the initial pH of the water used, which varied slightly in pH from 7.0 to 8.0. ΔH^+^ was calculated as the difference in microequivalents of H^+^ measured in the morning and the evening, with values above 0 indicating CAM activity. Student’s *t*-test was performed to check for ΔH^+^ values that were significantly different from zero, as well as to check for significant differences between watered and drought stressed samples on any given day.

### Leaf anatomy

Leaf thickness was averaged from five replicate leaves per plant, on one to two plants per genotype; the midrib was avoided, as were young or very old leaves. To measure succulence, the most recent, fully mature leaf per plant was collected, cutting just above the lighter-coloured petiole. Fresh leaves were immediately weighed, scanned, and dried in an oven at 60 °C. The final dry weight was recorded when the mass changed less than 0.01 g d^–1^. Leaf area was calculated in ImageJ (Rasband). Succulence was calculated as grams of water per centimetre of leaf area: (FW (g) – DW (g))/area (cm^2^), where FW and DW are fresh and dry weight, respectively. Genotype succulence values were calculated by averaging across replicate plants, and as residuals were not normally distributed, a Wilcoxon rank sum test was used to test for significant differences.

Leaf cross sections from all species (all individuals phenotyped with the LiCOR, plus additional genotypes; Supplementary Table S1) were prepared as follows: tissue was harvested from leaves, fixed in formalin, and embedded in paraffin. Samples were then sliced with a microtome, mounted, and stained with Toluidine blue by the University of Georgia Veterinary Histology Laboratory. Slides were imaged by a Zeiss microscope using ZEN software with one to three images taken per slide. Leaf anatomical characteristics were measured in ImageJ. All traits were measured independently on each image, then averaged across all images for a given genotype. Cell size was measured separately on adaxial and abaxial portions of the leaf and also averaged together for a cumulative average cell size. In many CAM plants, mesophyll cells fail to differentiate into palisade or spongy forms (Gibson, 1982), so these subdivisions were not taken into account when measuring cell sizes for either *Y. aloifolia* or *Y. gloriosa. Yucca filamentosa* does have clear mesophyll differentiation, and palisade and spongy mesophyll cells were used for adaxial and abaxial measurements, respectively. Leaf IAS was calculated by measuring the airspace in a given demarcated area of each cross section and dividing by the total area of mesophyll tissue in that area.

To address whether vein density is related to a plant’s use of CAM, vein spacing and the degree of 3D venation in the three species were measured. Average distances between major and minor veins were measured horizontally only when veins were clearly in the same plane and when xylem and phloem were visibly developed. The degree of 3D venation was assessed by counting the number of independent planes of veins (major or minor) in each cross section. Residuals of traits measured from cross sections were not normally distributed and the Wilcoxon rank sum test was used to assess species trait differences.

Stomatal density was measured using fresh tissue for the same genotypes used in gas exchange experiments many months after any treatment was imposed to avoid any effects of drought stress on stomatal density. In addition, leaves that were fully developed before treatment in the growth chamber were used. Leaves were painted with varnish on both adaxial and abaxial sides. The varnish was allowed to dry, then removed by adhering tape and gently removing the imprinted varnish. Images of stomatal peels were captured using the same Zeiss microscope and ZEN software as was used for cross sections. The stomatal density was calculated as number of stomata per area of epidermal tissue. An ANOVA was used to test for effects of species, side of leaf (upper or lower), and an interaction of the two. Neither the side of leaf nor the interaction of side with species was significant (*F*=1.174, *P*=0.286 for side of leaf; *F*=0.284, *P*=0.754 for the interaction of side×species), so these were not considered in further tests of species differences, which were evaluated with the Wilcoxon rank sum tests.

To assess the phenotypic space described by the traits measured, anatomical traits (with the exception of the number of planes of veins in the leaf, which was invariable) were combined with titratable acidity ΔH^+^ values and gas exchange data to perform a principle coordinates analysis (PCA). ΔH^+^ values were averaged for both clones of a genotype measured from day 1, as all plants were well watered. ΔH^+^ values were included from drought-stressed clones from day 7 as a measure of the ability of a plant to accumulate malic acid under drought stress. As a proxy for the ability to use the CAM pathway, the carbon uptake values that occurred at night during a given day–night cycle were summed, and divided by the total carbon uptake value (the sum of all CO_2_ uptake values measured for that plant across a 24-h period). While it excludes the carbon uptake that occurred in the 4h between measurements, those values are not likely to be largely different from the ones recorded, and the overall proportion calculated is an accurate estimate of relative contribution of night-time carbon uptake. On the same matrix of phenotype data, a Spearman rank correlation matrix with Holm–Bonferroni adjusted *P*-values was calculated. Each trait was also tested for bimodality using Hartigan’s dip test, as correlation results may be influenced by strongly bimodal distributions.

## Results

### Genetic diversity

**Fig. 1.**
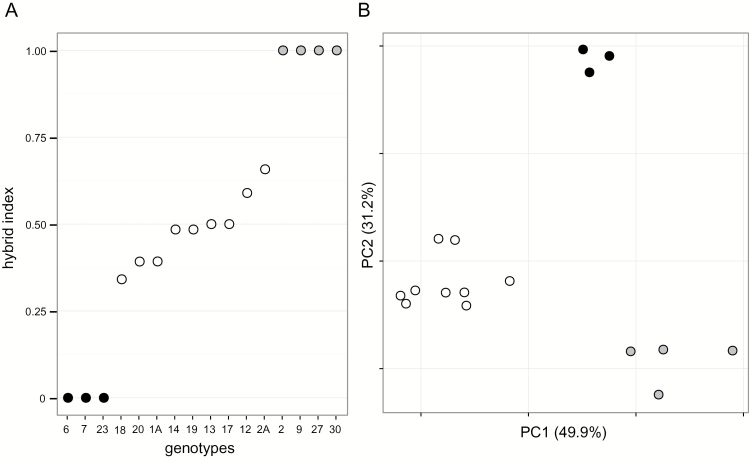
Microsatellite variation among genotypes represented by hybrid index scores (A), with parental scores set to 0 or 1 and the index value for the hybrid species representing proportion of ancestry from each parent, and PCA of distance matrix of multi-locus genotypes (B). *Yucca aloifolia* is represented by black filled circles, *Y. gloriosa* by open unfilled circles, *Y. filamentosa* by grey circles.

Hybrid index scores for the hybrid ranged from ~0.35 to ~0.65, with two individuals having an index of 0.5 (Fig. 1A). These two individuals had loci that were monomorphic for parental alleles, indicating they are not F1 hybrid genotypes. Hybrid indices indicate *Y. gloriosa* samples used in this study were largely later generational hybrids segregating for alleles from each parent. The first two principal components of the genetic distance PCA explain 81.1% of the variation among the three species (Fig. 1B). The species cluster into three distinct groups; there is no evidence of ongoing back-crossing of *Y. gloriosa* to either parental species in the samples used in this study. Rather, the marker data suggest that *Y. gloriosa* is distinct from both *Y. aloifolia* and *Y. filamentosa,* and is likely to be on an independent evolutionary trajectory from either parental species.

### Gas exchange pattern

**Fig. 2.**
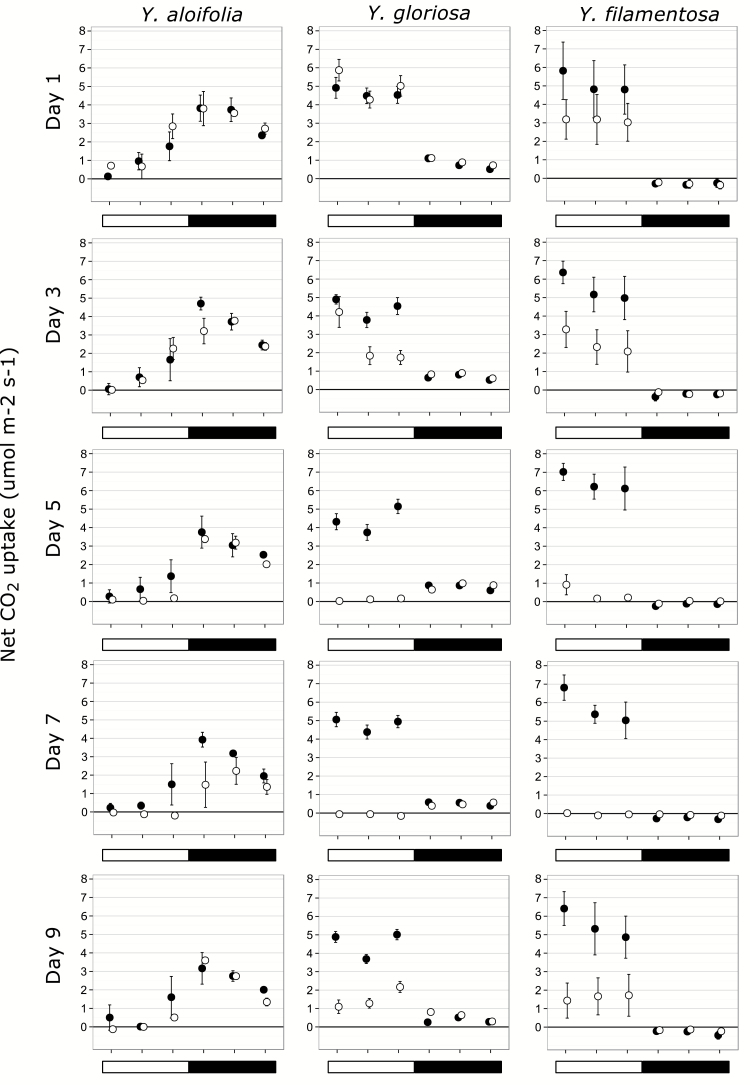
Gas exchange patterns for *Y. aloifolia*, *Y. filamentosa*, and *Y. gloriosa* across all days of the growth chamber dry-down. Filled circles indicate the clone kept under well-watered conditions, open circles indicate clones which were subjected to dry-down starting after day 1. The open bar indicates hours under light; the filled bar indicates time when lights were off. Points represent averages across all genotypes measured for a given species, with standard errors (too small to be visible for some points).

Both parental species behaved as their photosynthetic types would predict under well-watered and drought conditions. *Yucca aloifolia* showed predominantly night-time CO_2_ uptake along with late afternoon uptake (Fig. 2). This pattern remained under well-watered conditions for all five days. Under drought-stressed conditions, carbon still entered the leaves of *Y. aloifolia* at night but photosynthetic rates were reduced, and daytime uptake became negligible. For *Y. filamentosa*, well-watered plants showed no ability to take up carbon at night (Fig. 2). Drought-stressed *Y. filamentosa* plants likewise showed no transition to night-time uptake, and total photosynthetic rates were reduced to nearly zero by day 5 across all time points measured. *Yucca gloriosa* showed high levels of daytime carbon uptake, but night-time carbon gain happened under well-watered conditions as well (Fig. 2). As drought stress was induced in the hybrid, daytime uptake of carbon dropped to zero and net carbon gain occurred entirely in the dark, although absolute values were never as high in the hybrid as in *Y. aloifolia*. Upon re-watering, the drought stressed clones of each species began returning to their original, well-watered phenotype.

All genotypes of the parental species were consistent in the general pattern of gas exchange expected for each photosynthetic pathway. Although overall rates of photosynthetic activity may have varied between genotypes, the CAM and C_3_ patterns were maintained in all genotypes of *Y. aloifolia* and *Y. filamentosa*, respectively (Supplementary Figs S1 and S2). *Yucca gloriosa* had somewhat more variable responses to drought, though nearly all genotypes used night-time carbon uptake at low levels even under well-watered conditions (Supplementary Fig. S3). Some genotypes of the hybrid had a more pronounced level of night-time carbon uptake under drought, though most maintained well-watered levels even while stressed.

### Titratable acidity

*Yucca aloifolia* had the highest levels of acid accumulation (Table 1) as indicated by ΔH^+^ and was the only species to show a significant effect of drought on the degree of leaf acidity (day 7, Student’s *t*-test, df=8, *P*<0.001). No sample of *Y. filamentosa* ever required titration, demonstrating a complete lack of acid accumulation in this species. *Yucca gloriosa* had variable acid accumulation across time, with a species average value significantly greater than zero on days 1,7, and 9 but not 5 (Table 1). Some genotypes of *Y. gloriosa* had no acid accumulations (Supplementary Fig. S4), while *Y. aloifolia* had variable but positive accumulation across all genotypes and all treatments (Supplementary Fig. S4).

**Table 1.** Night-time acid accumulation (ΔH^+^, µequivalents g^–1^) derived from difference in dusk and dawn titratable acidity measurements, averaged across samples for all three species with standard error Averages for plants kept well watered for the duration of the experiment or under drought stress (which began on day 3, not sampled, and ended after day 7). Significance values (Sig) indicate species by treatment measurements that are significantly different from zero by Student’s *t*-test: ***P< 0.001, **P<0.01, *P<0.05, n.s., not significant.

**Species and conditions**	**Day 1**		**Day 5**		**Day 7**		**Day 9**	
	**ΔH** ^**+**^	**Sig**	**ΔH** ^**+**^	**Sig**	**ΔH** ^**+**^	**Sig**	**ΔH** ^**+**^	**Sig**
*Y. aloifolia*								
Well watered	144.2±16.0	***	106.7±16.2	***	133.5±14.6	***	125.5±7.3	***
Drought stressed	135.1±6.9	***	119.8±6.7	***	48.0±14.2	**	99.5±9.1	***
*Y. gloriosa*								
Well watered	2.4±0.97	*	0.44±0.26	n.s.	2.0±0.71	*	1.6±0.67	*
Drought stressed	3.7±1.4	*	1.79±0.87	n.s.	1.7±1.0	n.s.	2.4±0.77	**
*Y. filamentosa*								
Well watered	0±0	n.s.	0±0	n.s.	0±0	n.s.	0±0	n.s.
Drought stressed	0±0	n.s.	0±0	n.s.	0±0	n.s.	0±0	n.s.

### Leaf anatomy

**Fig. 3.**
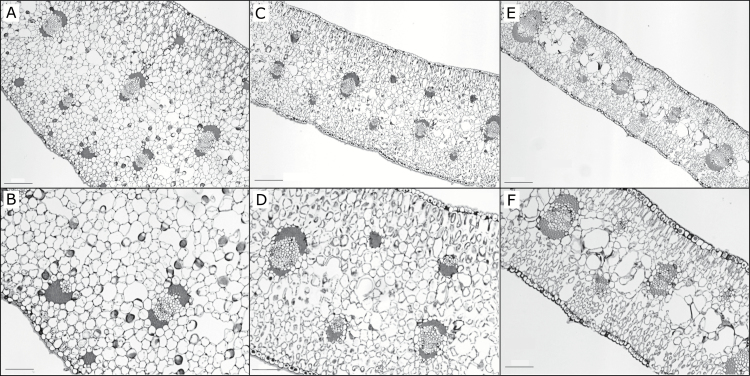
Cross sections of *Y. aloifolia* (A and B), *Y. gloriosa* (C and D), and *Y. filamentosa* (E and F) at 5× (A, C, E) and 10× (B, D, F) magnification. Scale bars for 5×=200 µm and at 10×=100 µm.

**Fig. 4.**
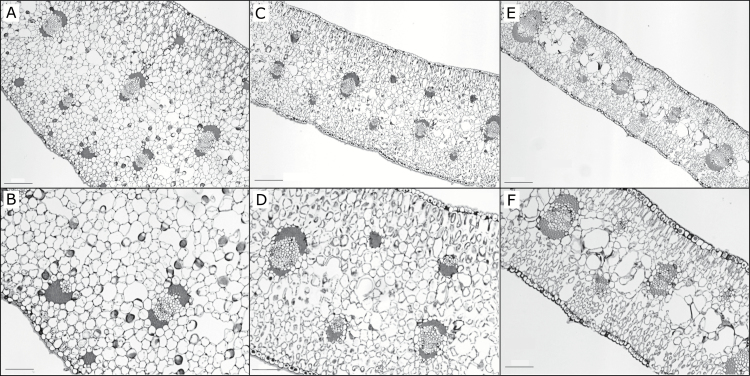
Intercellular air space vs. cell size, with standard errors around each genotype average. **Yucca* aloifolia* is represented by black filled circles, *Y. gloriosa* by open unfilled circles*, Y. filamentosa* by grey circles.

Cross sections of each of the three species were easily distinguishable (Fig. 3) and almost all anatomical traits measured were significantly different between all three species (Table 2). *Yucca aloifolia* had the most succulent leaves, followed by *Y. gloriosa,* with *Y. filamentosa’s* leaves being the least succulent. Leaf IAS was significantly smaller in *Y. aloifolia* and was the largest in *Y. filamentosa*. Similarly, *Y. aloifolia* had the largest cells. Across species, average cell size was negatively correlated to IAS (Fig. 4, *R* ^2^=0.4122, *P*<0.01), but this relationship did not hold within a species; in *Y. gloriosa*, for example, average cell size was not correlated significantly to IAS (*R* ^2^= 0.0737, *P*=0.42). However, there was no significant difference in the number of stomata on either adaxial or abaxial sides of the leaf between species, although *Y. gloriosa* has a weakly significant difference (*P*=0.04955) on the adaxial leaf surface compared to *Y. filamentosa.* The average distance between major veins was not significantly different between *Y. gloriosa* and *Y. filamentosa*, and *Y. gloriosa* did not differ in distance between minor veins from either parent, although the parental phenotypes were significantly different from each other. All three species showed similar propensities for 3D venation and there were no significant difference between them (Table 1).

**Table 2.** Mean and standard error for traits in the three species and significance of pairwise comparisons YA, *Y. aloifolia*; YF, *Y. filamentosa*; YG, *Y. gloriosa*. Sample sizes are 4, 10, and 7 genotypes of *Y. aloifolia*, *Y. gloriosa*, and *Y. filamentosa*, respectively, except for succulence, which had *n*=6, 12, and 8. IAS is shown as a percentage of mesophyll area. Stomatal and cell size values calculated separately for adaxial (ad) and abaxial (ab) surface areas. ***P< 0.001, **P<0.01, *P<0.05, n.s. not significant.

**Trait**	***Y. aloifolia***	***Y. gloriosa***	***Y. filamentosa***	**YA vs. YF**	**YA vs. YG**	**YG vs. YF**
Succulence (g cm^–2^)	0.1172±0.0027	0.0699±0.0022	0.0396±0.0022	***	***	***
Stomata, ad (mm^–2^)	75.02±5.78	125.11±17.14	79.06±5.60	n.s.	n.s.	*
Stomata, ab (mm^–2^)	93.06±13.07	125.12±17.15	92.88±8.63	n.s.	n.s.	n.s.
Thickness (µm)	1331.29±90.97	748.23±24.20	520.00±27.67	**	**	***
IAS (%)	14.5±0.54	21.5±1.28	28.7±2.38	**	**	*
Cell size, ad (µm)	2124.28±169.19	1153.99±95.21	696.31±47.85	**	**	***
Cell size, ab (µm)	1960.99±242.04	985.08±88.52	634.18±27.50	**	**	**
Dist major vein (µm)	701.56±34.37	535.59±37.08	450.86±61.73	*	*	n.s.
Dist minor vein (µm)	673.01±8.04	551.02±46.42	460.37±38.87	**	n.s.	n.s.
Planes of veins (µm)	2.46±0.30	2.55±0.19	2.55±0.14	n.s.	n.s.	n.s.

**Fig. 5.**
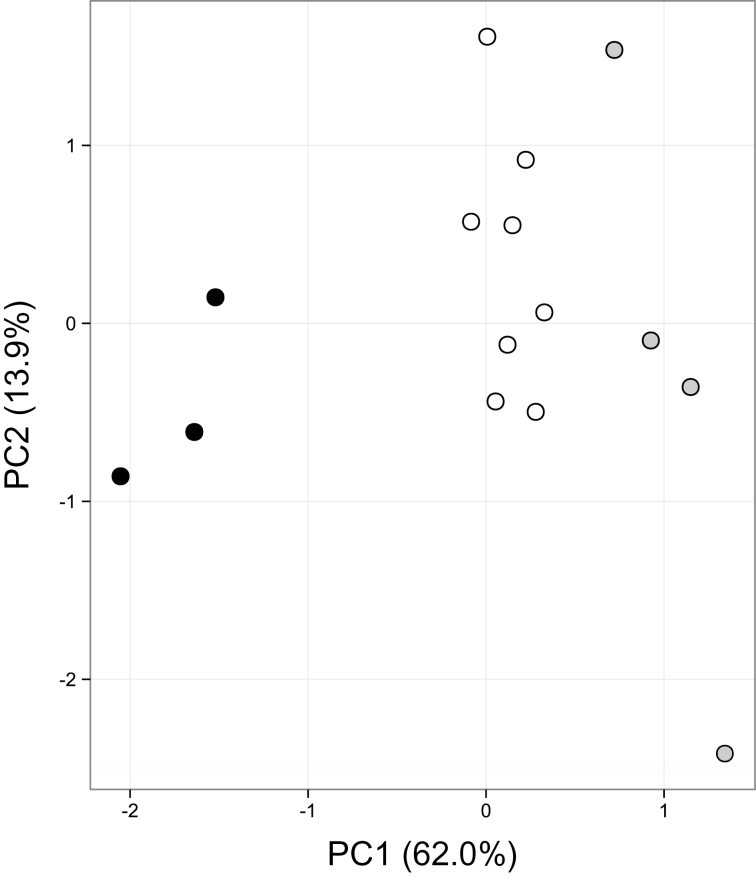
PCA of phenotypic data. *Yucca aloifolia* is represented by black filled circles, *Y. gloriosa* by open circles, and *Y. filamentosa* by grey circles. Phenotypes include those listed in Table 2: succulence, stomatal densities for adaxial and abaxial sides, leaf thickness, %IAS, average cell size on adaxial and abaxial sides, average distance between major veins, and average distance between minor veins, and additionally maximum dark CO_2_ uptake rate, maximum light CO_2_ uptake rate, ΔH^+^ well watered, ΔH^+^ drought conditions, and proportion of CO_2_ taken up at night well watered and under drought conditions.

**Fig. 6.**
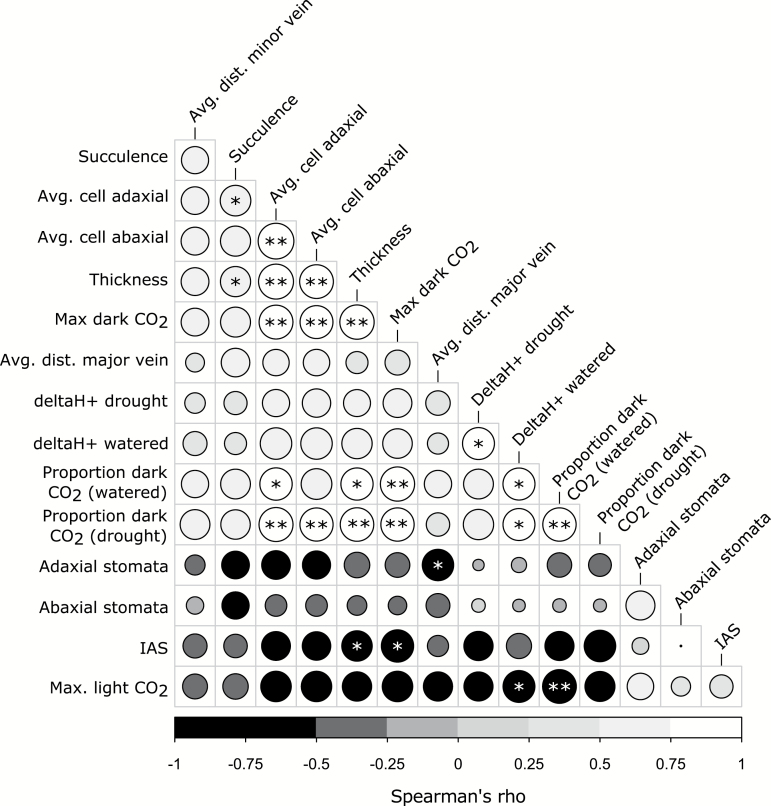
Correlation matrix of phenotypic data, based on Spearman’s rho. Rho of –1 is black, rho of 1 is white. The size of the circles indicates the absolute value of the correlation, with larger circles referencing larger absolute correlations. Holm-–Bonferoni corrected *P*-values: *P≤0.05, **P≤0.01. Trait names that are italicized on the vertical axis are traits that were not unimodal according to Hartigan’s dip test.

The PCA (Fig. 5) of all phenotypic data shows two ends of trait space defined by *Y. aloifolia* and *Y. filamentosa*. Further, *Y. gloriosa* appears slightly closer to *Y. filamentosa* but falls between the two clusters of parental species. The five traits with highest loading scores for the first principal component include adaxial and abaxial cell size (loading scores of –0.30 and –0.31, respectively), average proportion of dark CO_2_ uptake under watered conditions (–0.32), maximum dark CO_2_ uptake (–0.32), and leaf thickness (–0.31). The traits with highest loading scores for the second component include adaxial and abaxial stomatal density (–0.53 and –0.55, respectively), the average distance between major veins (0.44), succulence (0.22), and leaf IAS (0.21). The large loading scores for stomatal densities on PC2 are driven largely by a single genotype of *Y. filamentosa*. A number of traits were correlated, including positive relationships between leaf density (succulence, thickness, and cell sizes) and maximum dark CO_2_ uptake rates and proportion of CO_2_ taken up at night (Fig. 6). ΔH^+^ under watered conditions was positively correlated to proportion of dark CO_2_ uptake both under well-watered and drought conditions, but negatively correlated to maximum CO_2_ uptake in the light. Negative correlations existed between IAS and the maximum dark CO_2_ uptake rate, as well as IAS and leaf thickness. Only two traits—proportion of night-time CO_2_ uptake in watered and drought conditions—were not unimodal across species; correlations between proportions of CO_2_ uptake and any other trait should therefore be treated with caution.

## Discussion

### Photosynthesis in *Yucca*

Using a combination of gas exchange measurements, titratable acidity, and leaf anatomy, the use of the CAM and C_3_ photosynthetic pathways by *Y. aloifolia* and *Y. filamentosa*, respectively, was verified. These species of *Yucca* crossed to form a natural hybrid species, *Y. gloriosa* (Rentsch and Leebens-Mack, 2012), in which previous natural surveys showed nightly acid accumulation but no detectable nocturnal gas exchange (Martin *et al.*, 1982). Our results show that *Y. gloriosa* is an intermediate C_3_–CAM species, with the ability to uptake CO_2_ nocturnally but with relatively low levels of nightly acid accumulation (Fig. 2 and Table 1). Anatomically, the leaves of the three species are distinct, and *Y. gloriosa* has intermediate phenotypes for a variety of traits measured (Table 2). Trait values reported here for different *Y. gloriosa* genotypes are not only intermediate relative to the parental species, but also ‘fill’ the phenotypic space between the extremes of C_3_ and CAM. Individuals of *Y. gloriosa* sampled from natural populations are segregating for parental markers and none of the nine *Y. gloriosa* genotypes used in our analyses are F1s (Fig. 1). These results are in agreement with an earlier study suggesting that *Y. gloriosa* is genetically distinct from either parental species and is largely evolving independent of *Y. aloifolia* and *Y. filamentosa* (Rentsch and Leebens-Mack, 2012). The range of hybrid index scores for genotypes sampled in this and the earlier study raises the possibility that populations are segregating for CAM-related traits, but additional replication of individual *Y. gloriosa* genotypes is needed to assess intraspecific variation for physiological traits.

Gas exchange patterns for *Y. aloifolia* and *Y. filamentosa* are representative of their photosynthetic pathways, as are ΔH^+^ values. For *Y. gloriosa*, the photosynthetic machinery is intermediate; the hybrid has daytime CO_2_ uptake levels that are comparable to the C_3_ parent, and while it has the ability to use the CAM pathway at low levels, its night-time rate of CO_2_ uptake and acid accumulation never reached the levels in *Y. aloifolia.* Non-zero but low CO_2_ uptake rates at night are common in facultative CAM plants, as the facultative upregulation of the CAM cycle is often coincident with abiotic stress (Winter and Holtum, 2014). Since water stress decreased the magnitude of night-time CAM in *Y. aloifolia* (Fig. 2), it is unsurprising that drought stress likewise limited nocturnal CO_2_ uptake in *Y. gloriosa*. More important than the magnitude of night-time CO_2_ uptake is the proportion of daily carbon acquired at night relative to total carbon gain; for drought-stressed *Y. gloriosa* plants, 100% of carbon acquisition occurred during the night.

Acid accumulation in the hybrid was highly variable, though more acid accumulated in *Y. gloriosa* leaves than the C_3_ parent, paralleling its ability to use the CAM cycle at night. The lack of an increase in acidification under drought stress in *Y. gloriosa* is correlated to little increase in net CO_2_ uptake rates in drought-stressed plants relative to well watered. The variability among genotypes and between time points in acid accumulations (Supplementary Fig. S4) may be partly due to leaf heterogeneity and the inability to sample exact replicates in terms of leaf age and position. ΔH^+^ levels may also be so low in the hybrid that they were not detected consistently with titration methods used. Alternatively, the ability to accumulate acid in the leaves—a proxy for CO_2_ fixation by PEPC—may be segregating in genotypes of the hybrid, despite a relatively consistent ability across genotypes to acquire carbon via stomatal opening at night.

In species that are facultatively CAM, the use of the CAM pathway has been shown to relate to environment or seasonality (Guralnick *et al.*, 1984; Ball *et al.*, 1991; Borland *et al.*, 1992; Lüttge, 2006; Winter and Holtum, 2007). For example, in the annual *Mesmbryanthemum crystallinum,* induction of CAM occurs at the start of the dry season, with C_3_ photosynthesis being the primary mode of carbon acquisition during the wet period (Bloom and Troughton, 1979; Winter and Holtum, 2007). Similarly, *Clusia uvitana*, a weak CAM species, was shown to increase the proportion of carbon acquired via CAM from 27% in the wet season to 42% during the dry season (Zotz and Winter, 1994). In theory, seasonal or environmentally induced photosynthetic switching should allow plants to grow rapidly by using C_3_ photosynthesis during favourable conditions, and allow these plants to continue to grow by utilizing CAM during dry conditions or seasons. Even under extreme drought conditions, plants with CAM phenotypes can shut stomata completely and keep carbon metabolism primed by recycling respired CO_2_. This process, known as CAM ‘cycling’, produces no net growth as net CO_2_ intake is zero, but allows the photosynthetic machinery to stay active until conditions become more favourable. While drought stress forces *Y. gloriosa* from mostly C_3_ carbon gain to 100% CAM carbon gain, how this plant responds to drought in its natural setting is not known. *In situ* studies are required to better describe seasonal and environmental impacts on the frequency of CAM use in *Y. gloriosa*.

### Leaf anatomy

*Yucca aloifolia* possessed traits expected for CAM plants, including increased succulence and decreased IAS. Previous work in *Clusia, Annanas,* and *Kalanchoe* (Nelson and Sage, 2008) showed similar relationships between IAS and strong CAM. Zambrano *et al.* (2014) found that CAM *Clusia* species have thicker leaves, are more succulent, and have lower IAS than their C_3_ counterparts. The correlation between leaf thickness or succulence, internal air space, and CAM across different plant lineages indicates that these traits represent fundamental requirements for the CAM cycle. An increase in succulence, and the corresponding decrease in IAS, has been hypothesized to limit conductance of gases through the mesophyll. For CAM plants that generate very high concentrations of CO_2_ in the cells during daytime decarboxylation of malic acid, this limitation in conductance serves to keep captured carbon in the leaf. The intermediate levels of succulence and IAS found in *Y. gloriosa*, however, present a unique challenge to this species, as the majority of its carbon is assimilated during the day under well-watered conditions (Fig. 2). Movement of CO_2_ through the leaf is imperative for efficient C_3_ photosynthesis, which is typically inhibited by leaf CO_2_ levels. The proportion of IAS in *Y. gloriosa* measured here approaches levels found in the fully CAM *Y. aloifolia*, which would limit C_3_ photosynthesis during the day in *Y. gloriosa*. In addition, stomatal density was nearly identical in *Y. aloifolia* and *Y. gloriosa*, precluding the hybrid from using a greater number of stomata to compensate for lower leaf CO_2_ movement. How *Y. gloriosa* is able to conduct CO_2_ at the same level as *Y. filamentosa* could be addressed by stomatal aperture size, but the sunken nature and large subsidiary cells in stomata of *Yucca* make measuring aperture size difficult. In addition, IAS in *Y. gloriosa* is higher than in *Y. aloifolia*, and may conversely be limiting the hybrid’s ability to use the CAM pathway to as great a degree as is found in *Y. aloifolia*.

While research into the vascular architecture of CAM plants is lacking, a wealth of information about venation and photosynthetic efficiency comes from the C_4_ literature. An increase in leaf vein density is the basis of Kranz anatomy for C_4_ plants, enabling a high mesophyll to bundle sheath cell ratio required for efficient spatial concentration of CO_2_. For CAM, it is unlikely that vein density plays a direct role in photosynthetic efficiency as it does in C_4_ species; rather, the increase in succulence in CAM plants likely leads to modified venation patterns to maintain hydraulic connectivity. While increasing succulence is predicted to be correlated to a decrease in vein density and the distance between veins (Noblin *et al.*, 2008), succulent species have been shown to circumvent limitations to hydraulic connectivity by evolving 3D venation (Ogburn and Edwards, 2013). All three species of *Yucca*, including the C_3_ *Y. filamentosa*, had more than one plane of veins in their cross sections, indicating that the tendency toward 3D venation may be ancestral in *Yucca*, perhaps as a response to arid environments, and may have allowed for the further evolution of succulent CAM species in this genus (Griffiths, 2013). If propensities for thick leaves and 3D venation allow for repeated independent origins of CAM in a lineage, understanding why *Y. filamentosa* is not CAM will be important for describing the evolutionary trajectory from C_3_ to CAM.

### Evolutionary implications

Recent work on transitions from different photosynthetic states has focused on using intermediate plants that have varying propensities or similarities towards one state or another. *Flaveria*, which has C_3_, C_4_, and C_3_–C_4_ intermediate species, has allowed for detailed studies on the trajectory of anatomical, physiological, and genetic changes required to evolve C_4_ from C_3_ (Huxman and Monson, 2003; McKown and Dengler, 2007). Similarly, evolutionary studies within the grasses have described an anatomical progression from C_3_ to C_4_, and have shown that certain grass lineages possess pre-requisites for C_4_ photosynthesis (Christin *et al.*, 2013). While there are many evolutionary model systems in CAM plants, including facultative species in *Clusia, Mesembryanthemum, Kalanchoe*, and the Orhicdaceae, *Yucca* holds particular promise due to closely related species that are C_3_, CAM, and C_3_–CAM intermediates. In addition, molecular analysis has shown that individuals of *Y. gloriosa* are later generation hybrids that segregate for molecular markers (Rentsch and Leebens-Mack, 2012, this study); photosynthetic phenotypes may segregate as well, but this hypothesis needs to be tested. While this study does not have the necessary replication of genotype gas exchange patterns to conclusively shown variation in the ability to use CAM, leaf anatomical traits do differ between genotypes, and indicate that parental species physiology is likewise segregating among individuals of the hybrid. Future replicated analyses of gas exchange patterns across time points for more genotypes of *Y. gloriosa* will elucidate the degree of variation and genetic architecture of CAM-related traits within this species.

Finally, the intermediate nature of *Y. gloriosa* indicates it should be classified as a ‘C_3_–CAM species’ according to Winter *et al.* (2015) and raises the question of whether such intermediacy represents a stable state or a point on an evolutionary trajectory toward C_3_ or CAM. The traditional classifications of intermediate CAM include facultative species and CAM cyclers, although recent work in the Orchidaceae has described the prevalence of ‘weak’ CAM, a largely C_3_ plant with low levels of night-time acid accumulation (Silvera *et al.*, 2005). *Yucca gloriosa* does not fit into any of these traditional categories, and therefore prompts questions about the defining features of CAM plants (Winter *et al.*, 2015). Certainly, the ability of *Y. gloriosa* genotypes to obtain carbon entirely via the CAM cycle under drought stress should place it onto the CAM spectrum, but where along the continuum is less clear. Using *Y. gloriosa* and other intermediate species as models for evolution from C_3_ to CAM will help clarify the distribution of anatomical, physiological, and molecular traits along a CAM continuum. Better characterization of this continuum will ultimately inform understanding of CAM evolution and the potential for engineering CAM in a C_3_ species (Borland *et al.*, 2011, 2014; Borland and Yang, 2013).

### Conclusions

Assessment of photosynthetic pathway and leaf anatomy reveals *Y. gloriosa* as an intermediate C_3_–CAM species, possessing the ability to rely entirely on nocturnal carbon uptake during drought stress. This species’ ability to use CAM may be limited by a number of anatomical features, including smaller cells that prohibit accumulation of malic acid and greater intercellular airspace that might promote rapid loss of nocturnally fixed CO_2_ during the day. *Yucca gloriosa*’s intermediate nature poises it as an ideal system to study the evolution of CAM photosynthesis from a C_3_ ancestor, and future work will focus on understanding variation among hybrid genotypes.

## Supplementary data

Supplementary data are available at *JXB* online.

Table S1. Microsatellite primer sequences. 

Table S2. Locality information for all genotypes used in this study.

Table S3. Soil moisture probe measurements for % soil water content.

Figure S1. Values for gas exchange of all genotypes of *Y. aloifolia.*

Figure S2. Values for gas exchange of all genotypes of *Y. filamentosa.*

Figure S3. Values for gas exchange of all genotypes of *Y. gloriosa.*

Figure S4. Individual genotype values for leaf titratable acidity in all three species.

Figure S5. Raw correlation values between phenotypic traits shown in Fig. 6.

Figure S6. *P*-values for phenotypic traits shown in Fig. 6. 

Raw physiological data can be found at Dryad, doi:10.5061/dryad.pm4m5.

Supplementary Data
