# Supplementary material for: Gas exchange and leaf anatomy of a C3–CAM hybrid, Yucca gloriosa (Asparagaceae)
Source: J Exp Bot. 2015 Dec 29;67(5):1369–79. doi: 10.1093/jxb/erv536 (PMC4762382; doi:10.1093/jxb/erv536)
Supplement: Supplementary Data [file supp_67_5_1369__index.html]

Gas exchange and leaf anatomy of a C3–CAM hybrid, Yucca gloriosa (Asparagaceae) — Gas exchange and leaf anatomy of a C3–CAM hybrid, Yucca gloriosa (Asparagaceae) — Supplementary Data 

# Gas exchange and leaf anatomy of a C3–CAM hybrid, *Yucca gloriosa* (Asparagaceae)

## Supplementary Data

Data files

- Supplementary\_tables\_S1\_S3\_figures\_S1\_S6.pdf - Supplementary Data
