# Supplementary material for: Gas exchange and leaf anatomy of a C3–CAM hybrid, Yucca gloriosa (Asparagaceae)
Source: J Exp Bot. 2015 Dec 29;67(5):1369–79. doi: 10.1093/jxb/erv536 (PMC4762382; doi:10.1093/jxb/erv536)
Supplement: Supplementary Data [file supp_erv536_Supplementary_tables_S1_S3_figures_S1_S6.pdf]

Gas exchange and leaf anatomy of a C_3-CAM hybrid, *Yucca gloriosa* (Asparagaceae)

Karolina Heyduk, Nia Burrell, Falak Lalani, Jim Leebens-Mack

Table S1 – locality information for each genotype used in this study. Latitude and longitude reported in decimal degrees. States: NC = North Carolina, SC = South Carolina, GA = Georgia. Those designated with * were used for gas exchange measurement, and "GC block" indicates what date the clones were in the growth chamber.

Species	Genotype ID	GC block	State	Lat	Lon
*Y. aloifolia**	6	July 2014	SC	33.64912	-78.92852
*Y. aloifolia**	7	July 2014	SC	33.6501	-78.9256
*Y. aloifolia**	23	Feb 2015	GA	33.92746	-83.37906
Y. aloifolia	24	NA	GA	33.94787	-83.4092
Y. filamentosa	1	NA	NC	35.95847	-75.63843
*Y. filamentosa**	2	Oct 2014	NC	35.95818	-75.63808
Y. filamentosa	4	NA	NC	35.96482	-75.63393
*Y. filamentosa**	9	Oct 2014	SC	33.5044	-79.06227
Y. filamentosa	22	NA	GA	33.90708	-83.34247
*Y. filamentosa**	27	Feb 2015	GA	33.92129	-83.39032
*Y. filamentosa**	30	July 2014	GA	33.93197	-83.33298
*Y. gloriosa**	1	Feb 2015	GA	31.14714	-81.36619
*Y. gloriosa**	2	Oct 2014	GA	31.14633	-91.3664
*Y. gloriosa**	12	July 2014	SC	33.50278	-79.06448
*Y. gloriosa**	13	Feb 2015	SC	32.50592	-80.2931
*Y. gloriosa**	14	Oct 2014	SC	32.50398	-80.29545
Y. gloriosa	15	NA	SC	32.50393	-80.2957
*Y. gloriosa**	17	July 2014	SC	32.39087	-80.43033
*Y. gloriosa**	18	Oct 2014	GA	31.11907	-81.40818
*Y. gloriosa**	19	Oct 2014	GA	31.02172	-81.4348
*Y. gloriosa**	20	Feb 2015	GA	31.14895	-81.36608

Table S2 – Table of microsatellite primer sequences and repeat motif/length. Source indiciates where the primers were derived from: 2011 indicates a source of Flatz et. al 2011, *de novo* is a new SSR developed for this study, and 2012 is from Rentsch and Leebens-Mack 2012.

Name	Source	Sequence	Motif	Length
Yb12-forward<br>Yb12-reverse	2011	AACTCCCGTGTTTTGGTGTG<br>AACTCTACTGCCATGTATGTACGC	TACA	124-132
Yb04-forward<br>Yb04-reverse	2011	GCGCATTTTTGTTATTCTATGC<br>TCAGCAGCAACCGACAATAG	CT/GT	162-184
36-forward<br>36-reverse	2011	TACCCGTTCTTGCGGATAGT<br>GCTGAGTTCATCGTCGTCCT	CT	165-179
34690-forward<br>34690-reverse	*de novo*	CTGAGTGTGCTACACTTTTCC<br>CGTAAACATGATTTCTCATCC	CTTCAC	167-173
24908-forward<br>24908-reverse	*de novo*	GACGTAGCTGTCACAGATGTT<br>TTTCTTCGTTTCGTTAACTTG	CT	159-165
13-forward<br>13-reverse	2012	TTACCGAAGCCAGCTCTGC<br>GGAGTGAGAGAGGGAGTGG	AG	234-243
1-forward<br>1-reverse	2012	CCGACTTCCACCGAACTTG<br>AGACCCAGCGATGATGGAG	CAG	181-201

Table S3 – Soil moisture probe measures for % soil water content, taken every other day prior to first LiCor measurement with a soil moisture probe. Plants are indicated by species identifiers ("YA" = *Y. aloifolia*, "YF" = *Y. filamentosa*, and "YG" = *Y. gloriosa*) as well as their genotype designation (number/letter). Grey shaded rows are plants that were assigned to the drought stress treatment.

**Plant**	**Day1**	**Day3**	**Day5**	**Day7**	**Day9**
YA6.1	20.2	15.6	6.3	6.4	11.7
YA6.2	17.5	28.3	20.4	17.2	15.8
YA7.1	6.1	13.3	13.8	15.3	15.5
YA7.2	14.2	12.9	6.0	3.5	8.6
YA23.1	9.9	8.4	11.0	10.8	12.6
YA23.2	14.7	14.8	10.5	5.6	5.6
YF2.1	5.9	1.9	0.2	0	12.1
YF2.2	7.9	14.5	14.2	11	13.7
YF9.1	14.1	10.4	5.1	0.7	15.4
YF9.2	9.0	15.2	16.4	14.8	17.6
YF27.1	8.7	8.6	14.3	14.7	14.3
YF27.2	6.9	6.4	2.6	0	14.9
YF30.1	17.8	5.4	4.5	0.7	19.5
YF30.2	24.7	20.1	20.4	21.5	19.4
YG1A	12.5	9.5	25.1	16.9	19.7
YG1B	13.5	7.0	3.3	5.0	13.6
YG2A	12.3	7.4	2.1	0	17.2
YG2B	8.4	17.6	24.3	20.1	17.2
YG12.1	15.5	10.9	10.3	15.2	18.6
YG12.2	14.4	12.5	2.8	0	12.9
YG13.1	10.0	14.2	23.9	15.1	19.5
YG13.2	8.7	7.5	3.1	1.8	11.0
YG14.1	7.7	7.4	3.2	0	9.9
YG14.2	11.6	7.3	11.9	14.7	13.8
YG17.1	5.4	1.4	2.3	0.7	8.4
YG17.2	14.2	17	16	14.7	13.2
YG18.1	11.5	6.7	0.4	0	12.7
YG18.2	10.4	13.4	14.8	12.5	15.7
YG19.1	7.6	3.5	2.6	0.6	10.4
YG19.2	5.1	10.2	10.5	14.2	15.7
YG20.1	7.0	4.3	0.9	3.8	14.1
YG20.2	7.1	10.5	16.0	9.1	12.6

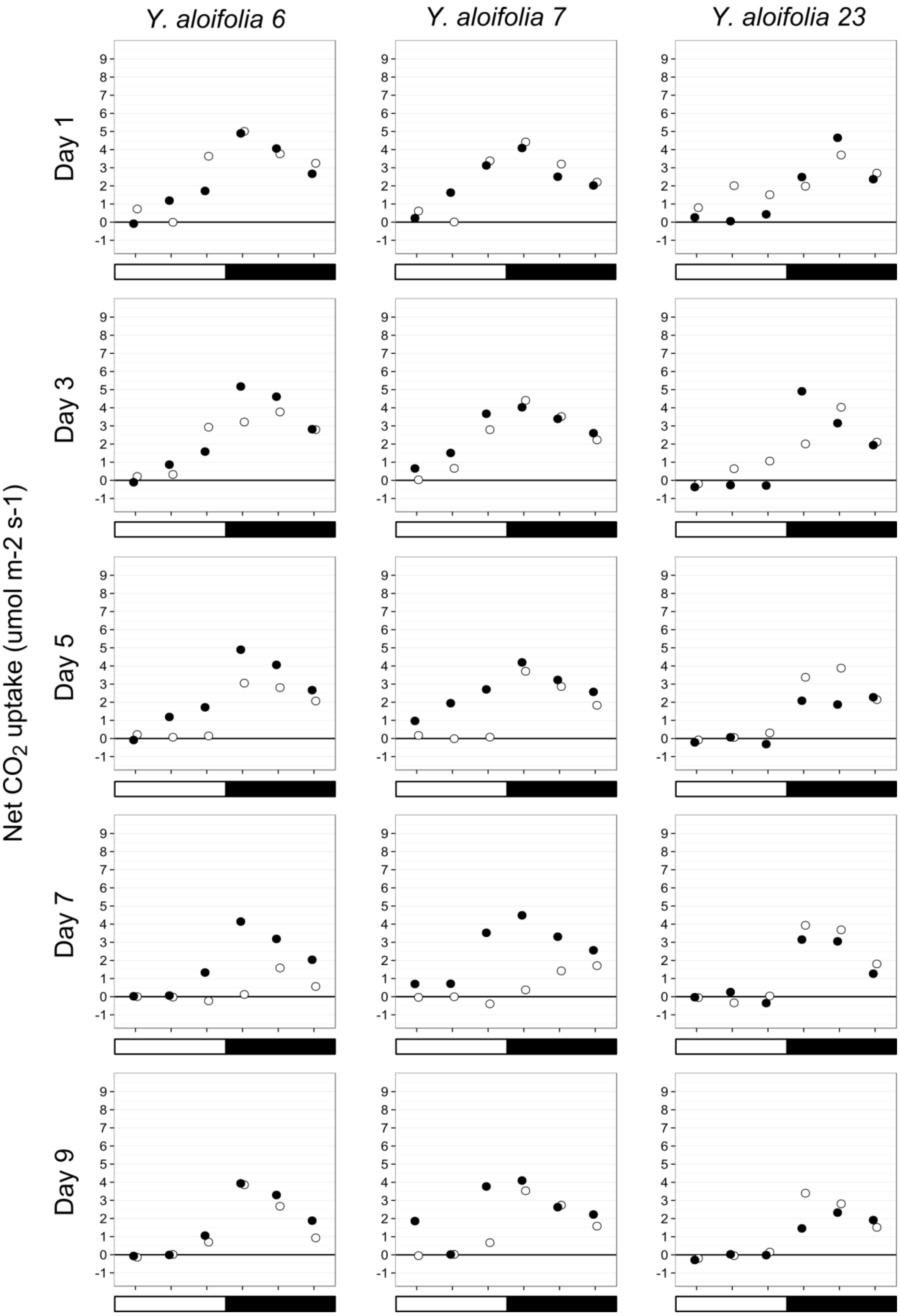

Figure S1 – Individual genotype gas exchange curves for the 3 samples of *Y. aloifolia*. Filled circles indicate the clone kept under well watered conditions, open circles indicate clones which were subjected to dry down starting after Day 1. The open bar indicates hours under light, the filled bar indicates time when lights were off.

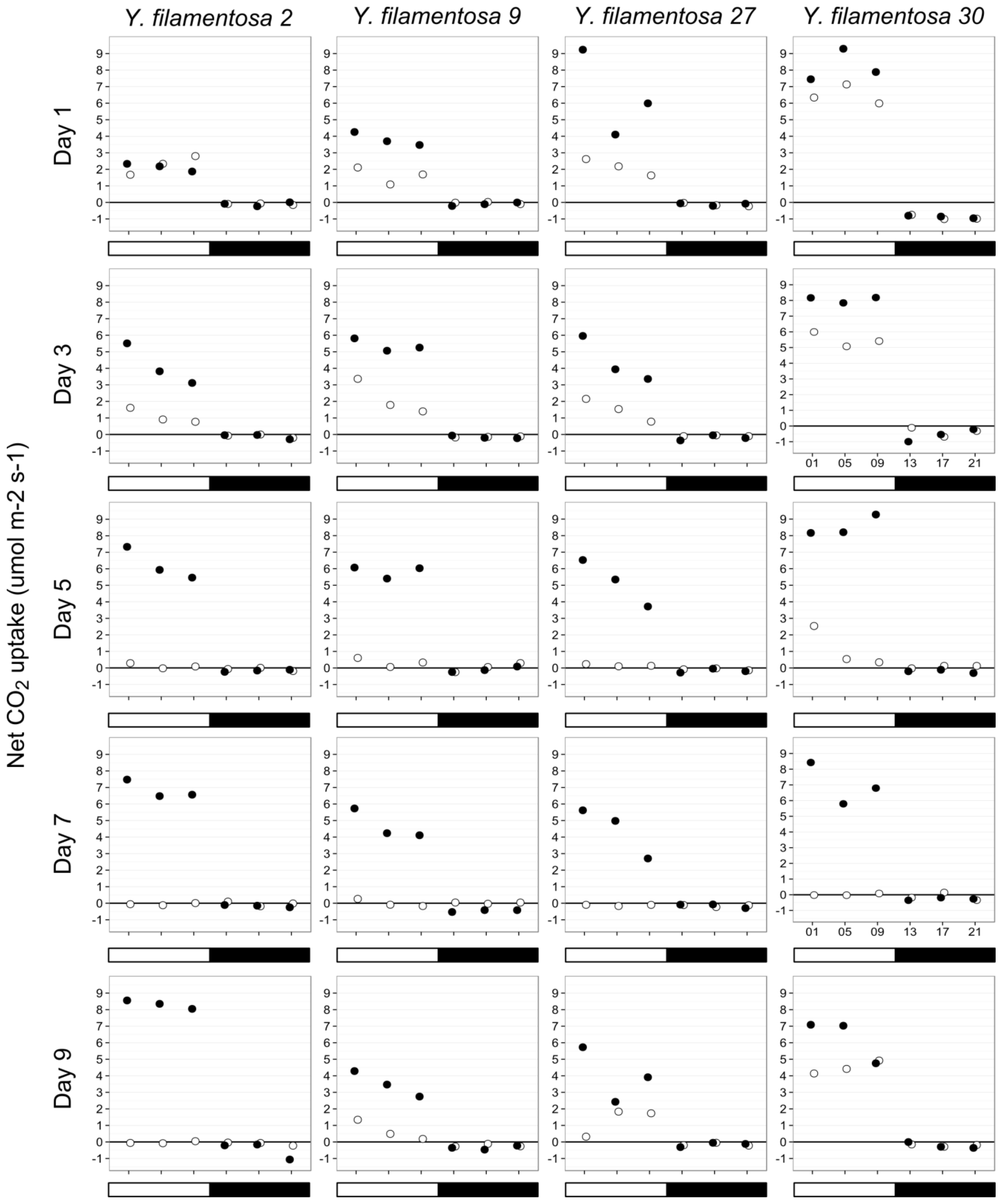

Figure S2 – Individual genotype gas exchange curves for the 4 samples of *Y. filamentosa*. Filled circles indicate the clone kept under well watered conditions, open circles indicate clones which were subjected to dry down starting after Day 1. The open bar indicates hours under light, the filled bar indicates time when lights were off.

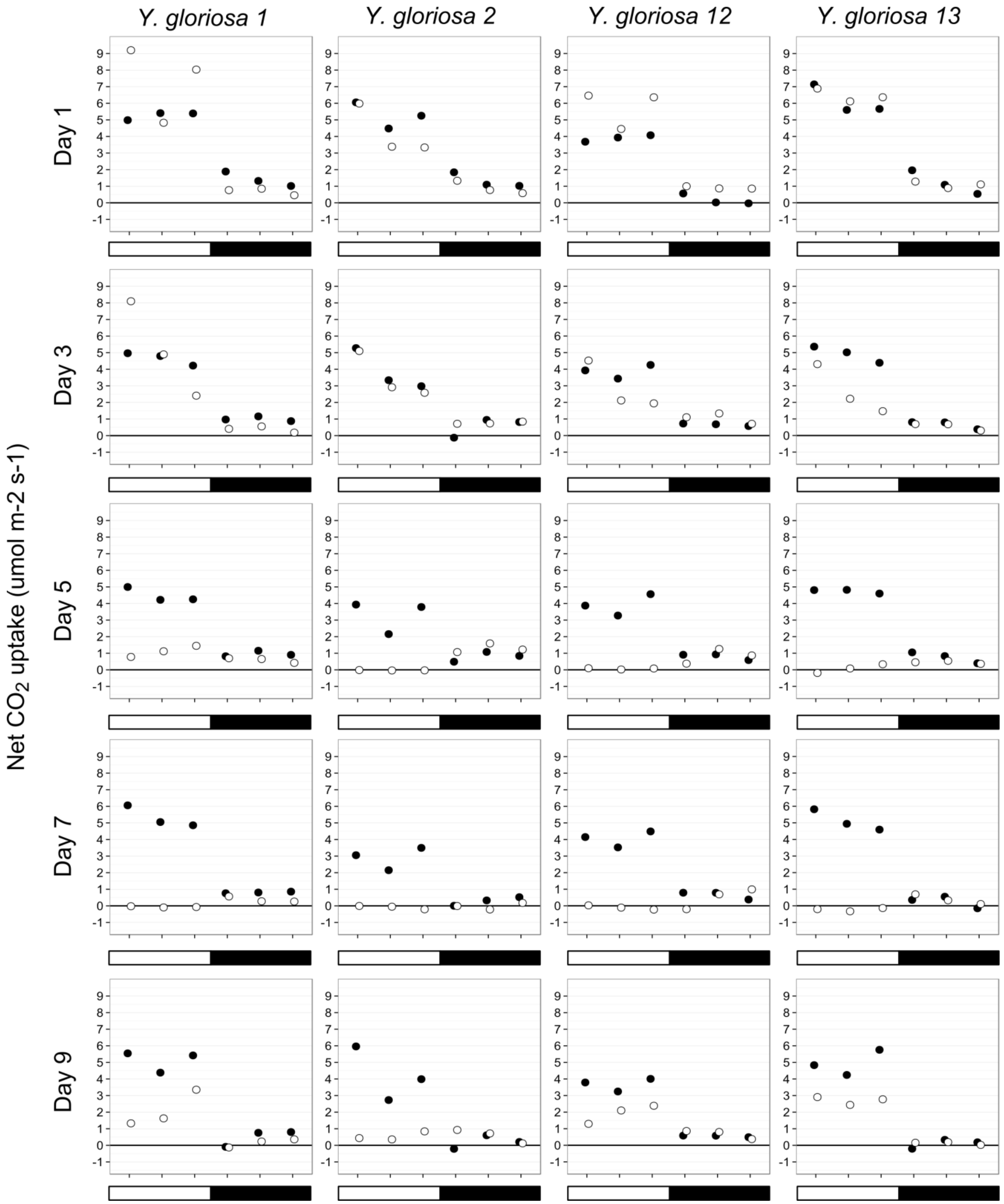

Figure S3 – Individual genotype gas exchange curves for the 9 samples of *Y. gloriosa*, divided into three panels. Filled circles indicate the clone kept under well watered conditions, open circles indicate clones which were subjected to dry down starting after Day 1. The open bar indicates hours under light, the filled bar indicates time when lights were off.

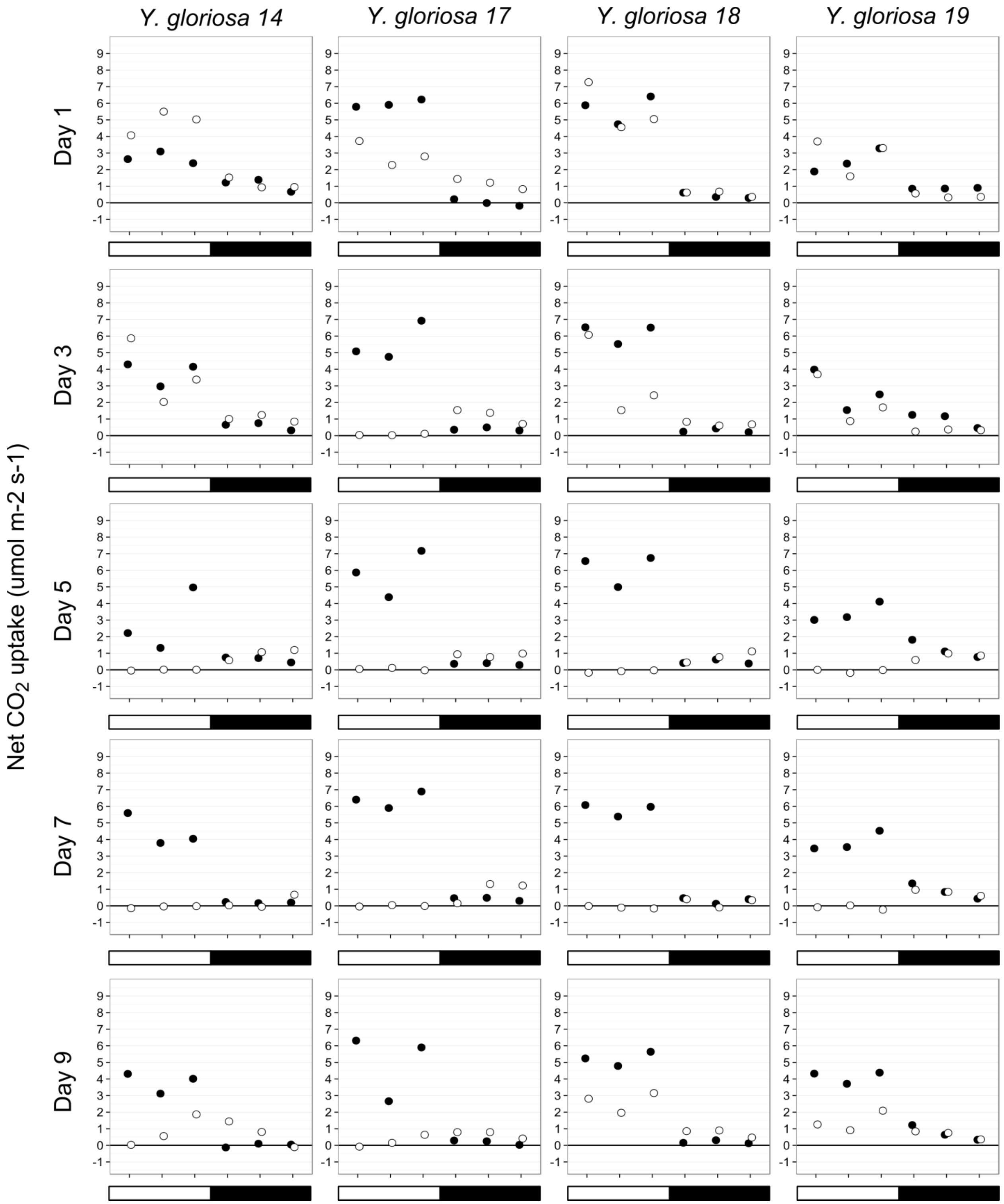

Figure S3 – Individual genotype gas exchange curves for the 9 samples of *Y. gloriosa*, divided into three panels. Filled circles indicate the clone kept under well watered conditions, open circles indicate clones which were subjected to dry down starting after Day 1. The open bar indicates hours under light, the filled bar indicates time when lights were off.

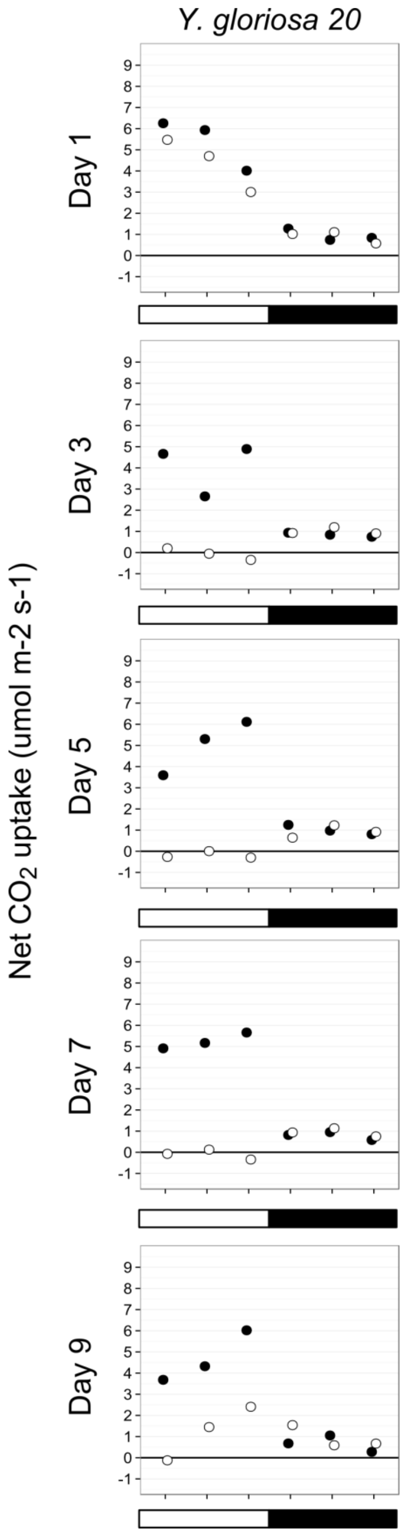

Figure S3 – Individual genotype gas exchange curves for the 9 samples of *Y. gloriosa*, divided into three panels. Filled circles indicate the clone kept under well watered conditions, open circles indicate clones which were subjected to dry down starting after Day 1. The open bar indicates hours under light, the filled bar indicates time when lights were off.

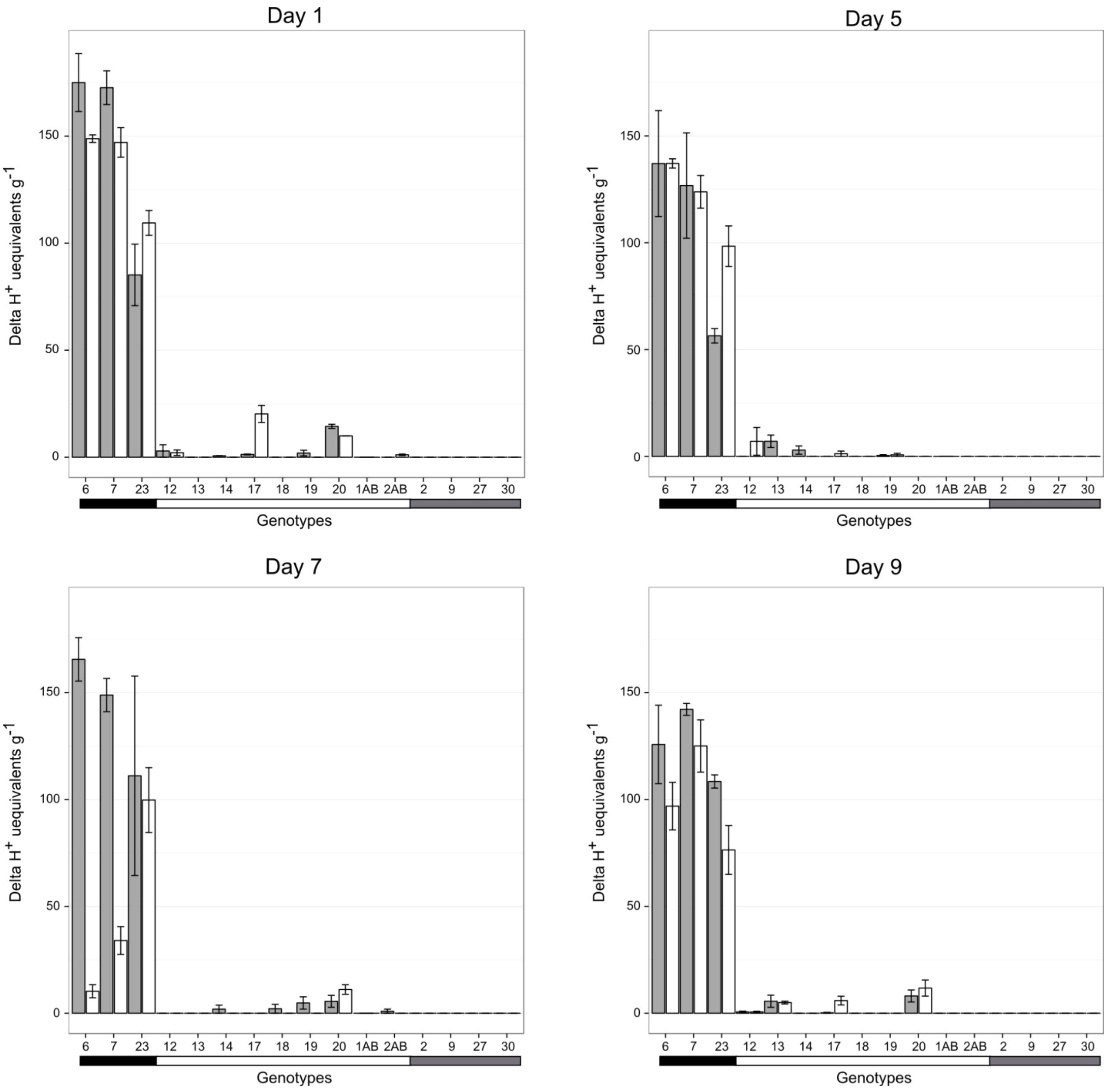

Figure S4 – Genotypic deltaH+ (H+ uequivalents AM – H+ uequivalents PM, per gram of tissue) values across the four days measured. Black line under genotypes are *Y. aloifolia*, open bar is *Y. gloriosa*, and grey bar indicates genotypes of *Y. filamentosa*. Grey vertical bars indicate measurements from watered plants (except day 1, where all plants were well-watered), open bars indicate plants in the drought treatment. Standard error reported with error bars.

Figure S5 – Raw Spearman correlation coefficients.

	Succulence	Adaxial stomata	Abaxial stomata	Leaf thickness	IAS	Avg. dist. Major vein	Avg. dist minor vein	Max. dark CO_2	Max. light CO_2	Proportion dark CO_2 (watered)	Proportion dark CO_2 (watered)	deltaH+ (w)	deltaH+ (d)	Avg. cell size adaxial	Avg. cell size abaxial
Succulence															
Adaxial stomata	-0.57														
Abaxial stomata	-0.55	0.63													
Leaf thickness	0.68	-0.49	-0.29												
IAS	-0.41	0.21	0.00	-0.69											
Avg. dist. major vein	0.61	-0.74	-0.43	0.37	-0.34										
Avg. dist minor vein	0.55	-0.30	-0.23	0.58	-0.43	0.27									
Max. dark CO_2	0.67	-0.46	-0.26	0.89	-0.78	0.47	0.58								
Max light CO_2	-0.49	0.53	0.28	-0.64	0.41	-0.64	-0.46	-0.70							
Proportion dark CO_2 (drought)	0.67	-0.45	-0.14	0.78	-0.63	0.56	0.56	0.88	-0.85						
Proportion dark CO_2 (watered)	0.65	-0.39	-0.13	0.84	-0.74	0.41	0.67	0.83	-0.67	0.84					
DeltaH+ (w)	0.37	-0.17	-0.12	0.69	-0.48	0.33	0.43	0.66	-0.76	0.77	0.77				
DeltaH+ (d)	0.40	-0.10	0.14	0.55	-0.64	0.45	0.32	0.62	-0.63	0.67	0.69	0.77			
Avg. cell size adaxial	0.70	-0.61	-0.35	0.92	-0.62	0.52	0.57	0.86	-0.70	0.79	0.87	0.75	0.55		
Avg. cell size abaxial	0.68	-0.59	-0.35	0.86	-0.61	0.53	0.58	0.81	-0.69	0.71	0.83	0.74	0.60	0.96	

Figure S6 – Uncorrected (below diagonal) and Holm-Bonferroni corrected (above diagonal) p-values for correlations described in Fig. S5.

	Succulence	Adaxial stomata	Abaxial stomata	Leaf thickness	IAS	Avg. dist. Major vein	Avg. dist minor vein	Max. dark CO_2	Max. light CO_2	Proportion dark CO_2 (watered)	Proportion dark CO_2 (watered)	deltaH+ (w)	deltaH+ (d)	Avg. cell size adaxial	Avg. cell size abaxial
Succulence		0.49	0.63	0.05	1	0.22	0.46	0.28	1	0.31	0.37	1	1	0.03	0.06
Adaxial stomata	0.01		0.25	1	1	0.03	1	1	1	1	1	1	1	0.32	0.41
Abaxial stomata	0.01	0		1	1	1	1	1	1	1	1	1	1	1	1
Leaf thickness	0	0.03	0.23		0.04	1	0.34	0	0.4	0.03	0	0.22	1	0	0
IAS	0.06	0.39	0.99	0		1	1	0.03	1	0.44	0.07	1	0.41	0.2	0.24
Avg. dist. major vein	0	0	0.07	0.1	0.14		1	1	0.41	0.99	1	1	1	0.67	0.65
Avg. dist minor vein	0.01	0.21	0.35	0.01	0.05	0.24		0.8	1	1	0.31	1	1	0.37	0.33
Max. dark CO_2	0	0.09	0.35	0	0	0.07	0.02		0.2	0	0.01	0.34	0.49	0	0.01
Max light CO_2	0.06	0.04	0.31	0.01	0.12	0.01	0.07	0		0	0.28	0.05	0.44	0.2	0.21
Proportion dark CO_2 (drought)	0	0.09	0.63	0	0.01	0.02	0.02	0	0		0.01	0.04	0.31	0.03	0.16
Proportion dark CO_2 (watered)	0.01	0.15	0.66	0	0	0.11	0	0	0	0		0.04	0.22	0	0.01
DeltaH+ (w)	0.16	0.54	0.68	0	0.06	0.21	0.09	0.01	0	0	0		0.04	0.07	0.08
DeltaH+ (d)	0.12	0.73	0.63	0.03	0.01	0.08	0.23	0.01	0.01	0	0	0		1	0.61
Avg. cell size adaxial	0	0.01	0.14	0	0	0.02	0.01	0	0	0	0	0	0.03		0
Avg. cell size abaxial	0	0.01	0.14	0	0	0.01	0.01	0	0	0	0	0	0.01	0	
